# Recent Developments in Plasmonic Nanostructures for Metal Enhanced Fluorescence-Based Biosensing

**DOI:** 10.3390/nano10091749

**Published:** 2020-09-03

**Authors:** Mohsin Ali Badshah, Na Yoon Koh, Abdul Wasy Zia, Naseem Abbas, Zahra Zahra, Muhammad Wajid Saleem

**Affiliations:** 1 Department of Chemical and Biomolecular Engineering, University of California-Irvine, Irvine, CA 92697, USA; 2 Plamica Labs, Batten Hall, 125 Western Ave, Allston, MA 02163, USA; nkoh1028@gmail.com; 3 Institute of Structural Health Management, Faculty of Civil Engineering and Engineering Mechanics, Jiangsu University, Zhenjiang 212013, China; abdul.wasy@ymail.com; 4 School of Mechanical Engineering, Chung-Ang University, Seoul 06974, Korea; naseem@cau.ac.kr; 5 Department of Civil & Environmental Engineering, University of California-Irvine, Irvine, CA 92697, USA; nzahra@uci.edu; 6 Department of Mechanical Engineering, University of Engineering and Technology, Lahore 54890, Pakistan; wajidsaleem@uet.edu.pk

**Keywords:** plasmonic nanostructures, metallic nanostructures, metal-enhanced fluorescence, localized surface plasmon resonance, low-dimensional materials, nanofabrication, biosensors

## Abstract

Metal-enhanced fluorescence (MEF) is a unique phenomenon of surface plasmons, where light interacts with the metallic nanostructures and produces electromagnetic fields to enhance the sensitivity of fluorescence-based detection. In particular, this enhancement in sensing capacity is of importance to many research areas, including medical diagnostics, forensic science, and biotechnology. The article covers the basic mechanism of MEF and recent developments in plasmonic nanostructures fabrication for efficient fluorescence signal enhancement that are critically reviewed. The implications of current fluorescence-based technologies for biosensors are summarized, which are in practice to detect different analytes relevant to food control, medical diagnostics, and forensic science. Furthermore, characteristics of existing fabrication methods have been compared on the basis of their resolution, design flexibility, and throughput. The future projections emphasize exploring the potential of non-conventional materials and hybrid fabrication techniques to further enhance the sensitivity of MEF-based biosensors.

## 1. Introduction

Nanostructures were investigated extensively over the past two decades due to numerous characteristics associated with unique phenomena that happen at the nano-size scale [1,2,3]. Localized surface plasmon resonance (LSPR) is one of the distinctive phenomena of nanostructures, where light produces strong oscillations of electrons when it interacts with the surfaces or structures of dimensions lower than its wavelength [4]. This unique phenomenon further involves localizing the light within the sub-wavelengths by breaking the light diffraction limit corresponding to dimensional features, which produces a strong localized electromagnetic (EM) field.

Metals have a proven history as materials for fabricating plasmonic nanostructures/nanoparticles with remarkable properties, including enhancement in photothermal/photocatalytic activity, surface-enhanced Raman scattering (SERS), and metal-enhanced fluorescence (MEF) [5,6]. Among said applications, enhancement in MEF is an area of particular interest due to its wide-range usability in photonics, medical diagnostics, and nanobiotechnology [7,8,9]. Variations in the type of materials, composition, and geometric design of nanostructures significantly affect the photodegradation resistance, fluorescence intensity, and fluorophores photostability [10,11].

Coinage metals like silver (Ag), gold (Au), and copper (Cu) are common materials used for MEF applications due to their desired characteristics, i.e., high reflection, electron conductivity, suitability, and biocompatibility [10,12,13]. However, many other materials such as aluminium (Al), palladium (Pd), and platinum (Pt), as listed in Table 1, were also investigated over the past decades to enhance MEF [14,15]. Table 1 enlists the plasmonic features and chemical reactivity of each material and refers to relevant investigations for detailed study. Ag and Au are materials widely reported for MEF-based applications due to their broad working range of wavelengths (visible (VIS) to near-infrared (NIR)) and high quality (Q) factor [10,16]. The Q-factor represents the strength of the surface plasmons generated over the material surface, which is a driving factor for enhancing the electromagnetic field and the MEF factor [16]. Therefore, the Q-factor is an essential criterion to determine the potential of a specific material used for MEF applications. Other materials, i.e., Pd and Pt, exhibit the spectral properties in the visible region with low Q-factor and high surface plasmon damping, thus their usability is limited due to their low MEF factor [14,15,16,17].

In addition to the materials and compositions, the MEF characteristics (i.e., intensity and electrons oscillation) critically depend on the shape and size of the nanostructures [7,10,20]. The size of the nanostructures governs the scattering and absorption ratios, active surface plasmon (SP) mode, the peak position of the plasmon mode, and localization of the plasmons [21]. Previous investigations [22] showed that the variations in size of the nanostructures can boost the MEF enhancement factor (MEF-EF). MEF-EF is defined as the ratio of fluorescence intensity between nanostructured and conventional glass substrates, measured at the same wavelength and under the same experimental conditions. The shape of the nanostructures is another critical parameter for describing the plasmonic characteristics [20]. Different shapes of nanostructures such as nanowires [10], spheres [13], rods [19,22,23], cubes [24,25], triangles [26,27], and crescent-like structures [28], were developed with multiple methods to tune the spectral properties, i.e., to boost the efficiency and MEF-EF [9,29,30].

Understanding and controlling the fluorescence properties in certain types of materials, as well as the materials’ nanostructures design and their optimization resulted in substantial improvement in many areas, from optoelectronics to biological sensing [9,21,29,30,31]. This article briefly describes metal enhanced fluorescence, its fundamental mechanism, and critically reviews the manufacturing methods to fabricate plasmonic nanostructures to enhance the MEF characteristics. Although many reviews have been published on surface plasmon resonance (SPR) sensing, this review focuses on morphology-dependent plasmonic nanostructures with MEF biocompatible platforms, in order to consolidate knowledge in its category.

## 2. Metal Enhanced Fluorescence

### 2.1. When Metal Enhanced Fluorescence Occur?

MEF is also known as plasmon-enhanced fluorescence or surface-enhanced fluorescence. It was first reported in the 1970s [32], and later adopted as a sensing technology, which was recognized in recent years due to the emergence of plasmonic nanostructures [9,33]. Various theoretical and experimental approaches have been published recently on MEF [30,34]. It is perceived that MEF occurs when fluorophores are excited near the metal surface at a distance ranging from 5 to 90 nm [34,35,36]. However, the fluorophores are quenched due to direct contact or being within the proximity (<5 nm) of the metallic surface. Thus, the interplay between the fluorophores distance and quenching effects either dominates or overwhelms the MEF enhancement factor [22,34]. Metallic nanostructures can enhance the fluorescence intensity to a scale of several hundred. However, there are inconsistent reports regarding the actual distance that delivers the maximum enhancement.

Similarly, numerous mechanisms of fluorescence enhancement have been reported; however, the precise mechanism is still debatable because of the complexity of the interactions between the metallic surface and the fluorophores. MEF is a complex phenomenon involving surface plasmon (SP) and the near optical field, which leads to enhancement in fluorescence intensity and photostability, but degrades the analyte life-span [37,38,39].

### 2.2. Metal Enhanced Fluorescence Mechanism

**Figure 1:**
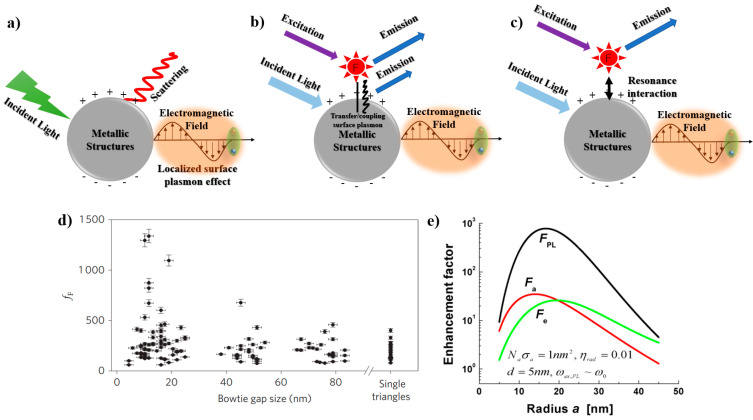
Metal enhanced fluorescence mechanism: (**a**) the localized surface plasmon resonance (LSPR) effect on metallic structures, (**b**) the plasmon coupling effect due to non-radiative interactions, modified from [47], (**c**) the intrinsic radiative decay effect, modified from [47], (**d**) fluorescence enhancement as a function of the bowtie structures gap size (adapted with permission from [50]), and (**e**) fluorescence enhancement (emissions, absorption, and total enhancement) as a function of the structure radius (adapted with permission from [53], Copyright 2009, Optical Society of America).

Considering the electromagnetic interactions, enhancement of MEF occurs due to several factors such as: (1) the localized surface plasmon effect, (2) the plasmon effect due to non-radiative interactions, and (3) the intrinsic lifetime [40,41]. As a first factor, the metallic structures or particles generate the LSPR effect, which strengthens the localized electromagnetic field, as shown in Figure 1a [8,42]. When incident light interacts with the metallic structures, it produces localized surface plasmon oscillations, which generate a highly concentrated electromagnetic field effect around the structures. This field-effect modifies the absorption characteristics of localized fluorophores by increasing their physical size [43,44], which results in enhanced fluorescence intensity due to their coupling with the nano-particles or -structures [45]. It is well-known that the geometry of the nanostructures is crucial in determining the MEF-EF. The fluorescence emission intensity from fluorophores increases under resonance condition due to the enhanced field effect at the edges and corners of the nanostructures, which enhances the MEF-EF [22,23,36,46].

As a second factor, non-radiative interaction mediates the SP coupling effect, as shown in Figure 1b [36,47]. This phenomenon results in excitation enhancement due to spectral overlap between the SP and fluorophores absorption band near or over the surface of the metallic nanostructures [40,41]. This overlap determines the dominant factor to be either Forster resonance energy transfer or the Purcell effect, and further, how this factor leads to quenching or fluorescence enhancement [48,49,50]. At the optimal distance, the fluorescence intensity is increased due to energy transfer between the SP and the fluorophores; this energy transfer is known as Forster resonance energy transfer (FRET). FRET is also known as a process of electron transfer through molecules [51,52]. Consensus is in place among researchers that the optimal distance between the metallic surface and fluorophores is critically important [34,35,36]. Recent theoretical and experimental studies have shown that non-radiative energy transfer not only depends on the EM field strength, but also on the spectral properties (i.e., absorption, scattering, emission) overlap, which leads to efficient fluorescence enhancement [37,47,48]. It is deduced that the fluorescence enhancement achieved within ~10 nm to metallic nanostructures surfaces, can be explained through FRET. The enhancement achieved through larger separations (10–50 nm) elucidates on the basis of the Purcell effect, which justifies the enhancement on the basis of controlled modification of the coupling between the light and matter [8,34,49]. The excitation enhancement is maximized using the metallic structures, which absorb light rather than scattering it, and with a concentrated electromagnetic field confined in narrow gaps or sharp edges of the metallic nanostructures, as shown in Figure 1d,e [50,53]. A few nanometers change in the geometry of the metallic structures significantly affect the excitation enhancement, which facilitates the development of MEF-based sensors with high sensitivity.

For the third factor of an intrinsic lifetime, the fluorophores and metallic nanostructures stay in close vicinity to each other. Therefore, the excited fluorophores couple together with the SP band of metallic nanostructures, generating new MEF decay pathways for energy transfer. The said phenomena facilitate the non-radiative energy transfer from the metallic nanostructures to fluorophores, allowing the fluorophores to excite and transmit energy to the far-field as radiative transfer, thus enhancing the fluorescence intensity (Figure 1c) [54,55]. The radiative rate of energy transfer from fluorophores can be modified by fine-tuning the metallic structures, which further decreases the fluorophore’s lifetime, due to the enhanced rate of radiative decay. In general, these coupling interactions correspond to the spectral overlap between SP and the emission band of the fluorophores. Therefore, quenching or a fluorescence emissions enhancement is possible depending upon the separation distance between fluorophores and metallic structures [47,53,54]. It is reported that if fluorophores are within a few nanometers of the metallic surface, the emissions will be quenched [44]. However, SP can still re-radiate the sufficient amount of quenched energy, which enhances the emission intensity [56,57]. However, this effect is minimized in the near-field region due to higher-order SP oscillations, which do not allow fluorophores to re-radiate; thus, the overall emission is quenched [56,57,58]. At larger distances where the Purcell effect dominates, it leads to an enhancement in the radiative rate rather than the free space. Whereas, if the SP scatters more than absorption, then it will lead to fluorophores emissions enhancements [50,53]. It is difficult to achieve a pure emissions or excitations enhancement because of the wider SP scattering and absorption lines relative to fluorophores and a limited Stoke’s shift of dye. Therefore, a balance is required to present the overall enhancement effect. In general, quenching dominates at a few nanometers scale, which evolves into a too large enhancement (10–1000X) at the range of 10 to 50 nm separation. Thereafter, there is a turn to normal fluorescence emissions as the distance between the nanostructures further increases [59,60,61].

The size of the nanostructures affects the SP spectral properties, i.e., absorption and scattering cross-sections. The SP absorption is dominant for nanostructures smaller than 20 nm, whereas, scattering dominates for larger dimensions and increases with the increase in the size of the structures. This behavior is defined as “ratio of scattering to absorption”, which is size-dependent and independent of the nanostructures aspect ratio [62]. Spectral properties are greatly affected by the shape of the nanostructures [63]. For example, anisotropic morphologies, i.e., nanorods, nanotriangles, and cubes reported for enhancement of the LSPR effect [64,65]. However, their performance is reported as: highest sensitivity for nanorods, followed by nano triangles, and then nanospheres [62]. The selection of the shape depends on the creation of “hot spot” regions, where the electromagnetic field is enhanced due to the localized SP effect, which concurrently enhances the fluorescence intensity.

### 2.3. Metal Enhanced Fluorescence from Plasmonic Nanostructures

#### 2.3.1. Conventional Plasmonic Nanostructures for Metal Enhanced Fluorescence

Since flat glass slides coated with metallic island films has demonstrated [66] MEF, MEF has had exceptional growth in biosensing, biotechnology, and bioimaging applications [9,67,68]. Metallic island films were commonly grown for fabricating MEF nanostructured surfaces due to their intrinsic characteristic of supporting the SPs in the visible (VIS) and near-infrared regions [12,21]. In addition to the conventional metallic island films, colloidal nanoparticles were used extensively over the years for MEF applications, including for cellular imaging or paper-based MEF substrates for one-time usage [69,70]. To achieve significant MEF enhancement, researchers fabricated different nanostructured surfaces, including nanotriangles, fractals, and cube-like structures using various materials [71,72,73]. In each case, the resulting nanostructures showed significantly higher enhancement than the planar island films.

#### 2.3.2. Recent Developments in Plasmonic Nanostructures for Metal Enhanced Fluorescence

The recent developments in nanostructure fabrication for MEF applications over the past 10 years have evolved for broadening the spectral range due to the need for MEF-based analyses. These analyses required a substantial overlap between the plasmon absorption band of the plasmonic nanostructures and the fluorescence excitation band of the fluorophores. Nanostructures have the potential to tune the spectral properties at a specific wavelength; most of the recent developments focus on tuning the plasmon absorption band over a broad spectral range (VIS to NIR) by utilizing different materials with different structural geometry and shapes, and by maintaining an optimal distance [64,74,75].

In this section, the recent developments in plasmonic nanostructures for enhancing the MEF factor are described, with details in further subsections. These sections highlight the different shapes and geometries of plasmonic nanostructures and their optimal enhancement. All the quantitative and deduced data have been adopted from the referred publications, with proper citations.

#### Metal Enhanced Fluorescence from Nano-Particles and Nanoclusters Fabricated by Chemical Synthesis Methods

**Figure 2:**
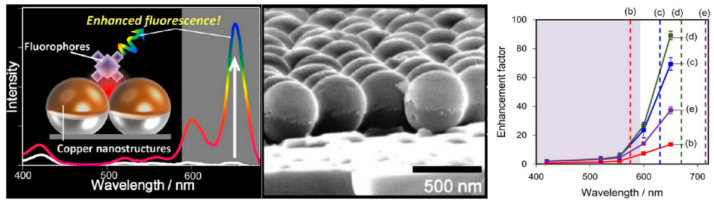
An ordered copper (Cu) nanosphere array along with the MEF enhancement factor (adapted with permission from Reference [13]).

Since the first attempt was taken to produce a MEF sensing platform using the Ag core–shell and silica core–shell particles [76], there has been an increasing demand to achieve an ideal MEF substrate for a better understanding of the MEF phenomenon with solution-based suspension methods. A summary of such structures with the MEF-EF is given in Table 2. It is reported that the MEF enhancement forms the metallic nanoparticles that critically depend on shape, interparticle distance, dielectric constant, and physical dimensions [7,57,77]. For metallic nanostructures/nanoparticles, the surface plasmon polarization (SPP) cannot exit, while the whole excitation happens because of strong LSPR. Hence, in nanoparticles- or nanocluster-based sensors, LSPR is used to enhance fluorescence by enhancing the fluorophores excitations or emissions [44,53]. In the case of enhanced excitations, the SP band overlaps with the fluorophores absorption band [78], and the separation distance should be minimized between the metal nanoparticles and the fluorophores [44,53]. In case of enhanced emissions, the SP band overlap with the fluorophores’ emissions band [59,60,61], and the separation distance should be maintained at around 10 to 30 nm [53]. Controlling these factors can significantly enhance the MEF performance for specific applications due to an enhanced electric field generated at the edges of the fabricated nanoparticles. Many researchers have performed studies to improve the MEF-EF by controlling the above-stated factors, especially the relationship between orientation and distance of fluorophores from the metallic surfaces [77,79,80]. Due to the coupling effect between the SPR band of the metallic nano-particles array surfaces and the fluorophores emissions, the fluorescence intensity from the nano-particles arrays was influenced by the distribution of the nanoparticles [81]. The metallic nanoparticles or clusters fabrication onto the substrate often generates randomly distributed “hotspots”, which help to attain a high fluorescence signal [60,82,83,84,85]. However, these structures have limited ability to achieve a high and uniform enhancement factor over a large area. Nanospheres, as depicted in Figure 2, fabricated by chemically synthesized silica spheres with thermally deposited Cu, show significant enhancement due to the tuning of LSPR modes [13]. It was found that the fluorophores’ quenching effect was enhanced at the longer wavelengths near the metallic surface, however with the fine-tuning of the nanostructures, the LSPR mode overcomes the quenching effect, and target molecules achieve an enhancement factor 89.2-fold compared with the reference substrate [13].

Recently, newly developed surfaces with anisotropic morphologies have gained more attention than the sphere-like morphologies due to their sharper pinnacles or vertices, which leads to the generation of stronger LSPR and local electromagnetic field effects [64,86,87]. In addition, these anisotropic structures also provide an opportunity to tune the wavelength over a wide range, from visible to near-infrared (NIR) [65]. For example, AuNRs, Ag, and AuNCs have rod-shaped, triangular-shaped, and nano-crescent structures, respectively. These structural features are used for tuning the LSPR characteristics over a wide range of wavelengths from visible to NIR, including the transparent biological window by adjusting the aspect ratio [28,88,89,90]. Therefore, anisotropic structures have great potential to be applied for constructing a highly sensitive MEF system for biological sensing.

**Figure 3:**
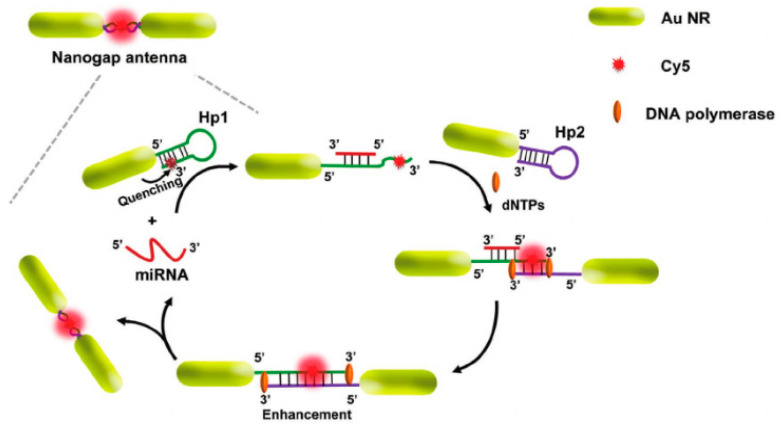
An illustration of the plasmon coupling and MEF enhancement due to end-to-end coupling and the distance effect (adapted with permission from Reference [88]).

Peng et al. [88] recently reported fluorescence enhancement from nanorod structures. Two DNAs were immobilized through their 5′ ends onto the edges of the nanorods, making the bond of Au-S, followed by the complementary target DNA immobilization, which was labeled with cyanine-5 (Cy5) as shown in Figure 3. The presence of nanorods demonstrated a large fluorescence enhancement when compared with a reference substrate without nanorods. This enhancement was attributed to the dual amplification phenomena. Firstly, there is an end-to-end coupling, which helps to tune and achieve an excellent spectral overlap at 660 nm between the LSPR band of AuNRs and Cy5 fluorophores; this further provides the opportunity for fluorescence enhancement due to a “hotspot” region, which typically occurs at the corners. Secondly, the DNA strand displacement helps to overcome quenching. Nanorods conjugated with fluorophores have also been reported as dual-modal nanoprobes for MEF and SERS enhancement [84]. Despite this, the dual-modal performance of nanorods structures was reported with an MEF-EF of only 2.2. There could be two possibilities for the lower MEF-EF, either: (1) inactive spectral overlapping of the plasmon and fluorophores absorption band, as the LSPR band generated at 510 nm, or (2) the growth of specific fluorophores dimeric species on the surface of the nanorods.

**Table 2:** Summary of various nanostructures and nanoparticles fabricated via chemical synthesis methods with different shapes and geometries, and their experimentally determined enhancement factor (EF), with feature size, excitation wavelengths, used fluorophores, and publication information such as year and reference, for further reading.

Material	Configuration of Structures	Structures Feature Size (nm)	Wavelength λ (nm)	Fluorophore	EF	Year	Ref.
Au	Nanocomposite	Dia: 20 nm	375 nm	Amantadine hydrochloride	1.4	2018	[91]
Au	Nanorods	Dia: 17 nm Length: 43 nm	532/785 nm	Rhodamine B	2.2	2012	[84]
Au	Nanoshells	Dia: 200 nm	760 nm	Rhodamine 610	2.4	2011	[92]
Au	Nanoparticle	Dia: 33 nm	450 nm	PPQ-Zn^2+^-PPQ	3	2019	[41]
Ag@SiO2@PMOs	Nanocubes	Dia: 50 nm	465 nm	Cu^2+^	3	2016	[93]
Ag	2D nanoparticle arrays	Dia: 20 nm	532 nm	Rhodamine 6G	3	2012	[94]
Ag@SiO_2_-Au	Nanoclusters	Dia: 50 nm	610 nm	AuNCs	3.2	2019	[95]
Au@SiO_2_-NH_2_@Au	Nanoclusters	Dia: 99 nm	610 nm	AuNCs	3.7	2018	[60]
Au	Nanorods	-	753 nm	Cy7	4.36	2020	[96]
Au	Nanobipyramids	-	751 nm	Cy7	5.63	2020	[96]
Ag	Colloidal nanoparticles	Dia: 123 nm	560 nm	Rhodamine 700	7	2019	[97]
Ag	Nanowires on template	Pores Dia: 200 nm	550 nm	Rhodamine B	7.5	2018	[98]
Ag	3D nanoparticle arrays	Dia: 20 nm	532 nm	Rhodamine 6G	8.5	2012	[94]
Au@SiO_2_	Core-shell nanoparticles	Dia: 89.7	642 nm	Alexa	9	2020	[99]
Ag/Au@Silca	Nanoclusters	Dia: 37 nm Thickness: 13 nm	635 nm	Cy5	9.4	2010	[100]
CuNCs	Nanoclusters	Dia: 40–50 nm	574 nm	CS-GSH-CuNCs	10	2020	[101]
Ag@SiO_2_	Nanoparticle	Dia: 90 nm	370 nm	Au_25_	12	2017	[102]
Ag	Nanoshells	Dia: 5 nm	420 nm	Rhodamine 123	20	2010	[103]
Ag/Au	Nanocluster	Dia: 25 nm	548 nm	Cy5	35	2010	[82]
Au	Nanorods	Dia: 13 nm	635 nm	Cy5	40	2010	[85]
Ag@SiO_2_	Core-shell nanoparticles	Dia: 89.7	642 nm	Alexa	70	2020	[99]
Cu	Nanospheres	Dia: 462 nm	650 nm	Porphyrin	89	2013	[13]
Ag	Nanoshells	Dia: 50–80 nm	514.5 nm	Rhodamine B	94	2012	[83]
Au	Nanorods	Dia: 18.1	760 nm	streptavidin-CW800	100	2018	[104]
Au	Nanocluster	Dia: 20 nm	365 nm	Eu^3+^-EUTC	100	2014	[105]
Ag@Au	Naprisms	-	532 nm	Ir-Zn_e_	110	2017	[89]

#### Metal Enhanced Fluorescence from Non-Periodic Nanostructures Fabricated by Deposition Methods

**Figure 4:**
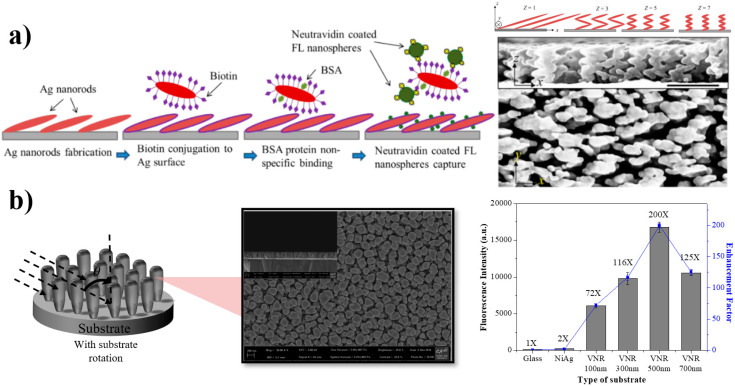
Examples of the MEF substrate fabricated by deposition methods: (**a**) a zigzag nanorods MEF array, and (**b**) a vertical nanorods MEF array. Adapted with permission from references [22,106].

This section describes another category of MEF nanostructures which have been significantly practiced over the past decade due to their inherent characteristics of time-efficient, economical, and large-area fabrication with the ability to tune the spectral properties [17,22,23]. MEF enhancement critically depends on the morphology of the nanostructures. Compared to other types of structures, deposition methods enable control over size, porosity, and importantly, the shape of the nanostructures, by manipulating the operating parameters, such as evaporation time, deposition rate, and incident angle [17,106,107]. Using the deposition methods, various functional coinage metals, i.e., Cu, Ag, Au, and Al, were used as building blocks to construct the numerous structures for enhancing the MEF-EF as summarized in Table 3. Anisotropic thin-film nanorods-like structures were fabricated by oblique angle deposition (OAD), to improve the MEF enhancement factor. Ju et al. [108] and Dhruv et al. [109] fabricated slanted nanorod structures by OAD and they studied the suitability of the structures for MEF applications. Ji et al. [106] fabricated zigzag structures, as shown in Figure 4a, by employing OAD, and reported a 28-fold EF for Alexa 488 detection with a 0.01 pM detection limit. Although anisotropic nanorods made with other methods were previously reported [85,89], Ji’s work has put forward a pathway for deposition experts in fabricating the MEF-based biosensor using deposition methods. The plasmonic response from such structures can be tuned by controlling the size of nanostructures, which helps to overlap the SP characteristics with the excitation of fluorophores. Recently, Badshah et al. [22] fabricated vertical nanorod structures, as shown in Figure 4b, by using glancing angle deposition (GLAD) and studied their feasibility for MEF applications. It was reported that increasing the length of the vertical nanorod structures changes the morphology of the nanorod structures. They also reported a 200-fold MEF-EF on the nanostructured surface (diameter: 120 nm, and length: 500 nm) using Cy5 fluorophores, compared to a reference substrate [22]. The researchers believed that the “illuminating-rod effect”, due to the enhanced electromagnetic field and the LSPR effect, might be the main contributor for enhancing the MEF-EF, along with the separation distance (20–30 nm) between the nanostructured surface and the DNA-conjugated fluorophores [22,80]. It was also found that the controlled porosity, diameter, and length of the nanostructures contribute significantly to enhance the MEF enhancement factor [22,109].

Although significant MEF performance is reported from all these fabricated structures, it is not possible to predict the performance of the structures precisely through theoretical modeling. The validity of these nanostructures needs additional experimental investigations to show exceptional enhancement in MEF and structural optimization, which is not a systematic approach to tune the spectral properties.

#### Metal Enhanced Fluorescence from Periodical Nanostructures Fabricated by Lithography Methods

Modern lithography is a powerful tool to fabricate periodic metallic nanostructures that can be tailored for efficient MEF studies to understand the underlying MEF concept. Irrespective of the type of structure, lithographic methods have the advantage of fabricating periodic structures, which can be utilized to map or predict the MEF performance with efficient process control [18,26,50]. A summary of such periodic structures with their MEF-EF is arranged in Table 4. The nano-prisms or nanotriangles fabricated by e-beam lithography has demonstrated a 33-fold MEF enhancement by controlling the feature size [18]. Levene et al. [116] reported on the fluorescence-based detection of single-molecule DNA in low volumes (10^−18^–10^−21^ L) inside a zero-mode waveguide (ZMW) consisting of holes array structures fabricated by e-beam lithography. This arrangement enables them to be adopted as a commercially available platform (Pacific Biosciences) for single-molecule DNA detection with real-time sequencing [117]. Although ZMWs fluorescence-based platforms gained recognition, the standard platform has the potential to be improved by robust optimization. Al is a known plasmonic material with low electromagnetic field enhancement; different structure shapes in conjunction with other plasmonic materials (Au or Ag) can further enhance the electromagnetic field and fluorescence. Recently, Paolo et al. [118] have reported the bi-metallic (Au–Al) nano-slots structures with improved sensitivity. It was reported that the bi-metallic nano-slots structures enhanced the fluorescence by 30-fold compared with the standard ZMW platform. In a similar study, researchers have fabricated bowtie nanoantenna structures by e-beam lithography and reported [50] 1340-fold MEF-EF with low quantum yield of N,N^1^-bis(2,6-diisopropylphenyl)-1,6,11,16-tetra-[4-(1,1,3,3-tetramethylbutyl)phenoxy] quaterrylene-3,4:13,14-bis(dicarboximide) (TPQDI) dye. A similar MEF enhancement was reported [119] with an EF of 1100-fold using the nanoantenna-in-box platform fabricated by focused ion beam milling (FIB). In spite of the promising results, the widespread implications of the e-beam lithography process are limited because of the difficulty in producing the nanostructures over a large area.

**Figure 5:**
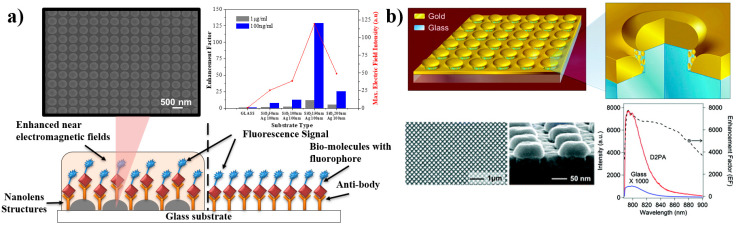
Examples of MEF substrate fabricated by nanoimprint lithography: (**a**) a plasmonic nano-lens array, and (**b**) a disk-coupled dots-on-pillar antenna-array (D2PA) for MEF. Adapted with permission from references [30,122].

Nanoimprinting has the advantage of producing periodic structures over a large area, with homogeneity. Recently, a plasmonic nano-lens array, as shown in Figure 5a was fabricated by nanoimprinting. The developed structures demonstrated a 128-fold MEF-EF for a biomolecule streptavidin conjugated with Cy5, by controlling the inter-lens spacing [30]. Recently, ZnO-nanorods structures with an Au layer were reported to have an EF of ~300-fold [120]. It was deduced that the optimized geometry of the ZnO structures enhanced the electromagnetic field. At the same time, the Au layer above the nanorods helps to reduce the absorption and results in enhanced emission [120]. “Disk-coupled dots-on-pillar antenna array” (D2PA) structures, as shown in Figure 5b, reported from the Stephen chou group at Princeton University, have demonstrated 2970-fold [121] and 7400-fold [122] MEF-EF for detection of immunoassays of Protein A and Immunoglobulin G (IgG), respectively. The team has also reported 4 × 10^6^-fold promising enhancement with a single fluorophore located in the proximity of the “hotspot” region [122]. The fluorescence enhancement occurred due to the generation of a highly confined electromagnetic field induced by the SP, localized within the “hot-spots”, which results in enhanced excitation of fluorophores and therefore increases the fluorophores radiative decay rate, which further enhanced the fluorescence.

**Table 4:** Summary of various nanostructures fabricated via various lithography methods with different shapes and geometries, and their experimentally determined enhancement factor (EF), with feature size, excitation wavelengths, used fluorophores, and publication information such as year and reference, for further reading.

Material	Configuration of Structures	Structures Feature Size (nm)	Wavelength λ (nm)	Fluorophore	EF	Year	Ref.
Ag	Nano triangles	Dia: 300 nm	525 nm	Alexa 488	7.8	2013	[26]
Ag	Concentric gratings	Width: 200 nm Height: 65 nm	635 nm	Alexa 647	10	2011	[123]
Ag	Nano gratings	Pitch: 300 nm	532 nm	Rhodamine 6G	14	2011	[124]
Al_2_O_3_@Ag	Nano gratings	Dia: 142 nm Height: 67 nm	400 nm	Rhodamine 6G	14	2016	[125]
Ag	Nanodots	Dia: 100 nm Height: 30 nm	560 nm	Cy3	15	2011	[126]
Au	Nanocylinders	Dia: 100 nm Height: 35 nm	580 nm	CdSe/ZnS core shells	26	2006	[18]
Ag	Nano gratings	Pitch: 375 nm	532 nm	Rhodamine 6G	30	2011	[124]
Au	Nanoprisms	Width: 100 nm Height: 35 nm	580 nm	CdSe/ZnS core shells	33	2006	[18]
Au	Nanogaps	Height: 60 nm Pitch: 400 nm	670 nm	Cy5	47.4	2014	[127]
Ag	Nano triangles	Dia: 500 nm	780 nm	Alexa 790	83	2013	[26]
Ag	Nano gratings	Height: 44 nm Pitch: 400 nm	530/550 nm	Rhodamine 6G	116	2015	[128]
Ag	3D nanodomes	Dia: 250 nm Height: 100 nm Pitch: 500 nm	635 nm	streptavidin-Cy5	128	2018	[30]
Ag	3D nano gratings	Height: 30 nm Pitch: 480 nm	632.8 nm	Cy5	170	2017	[129]
ZnO	Nanorods	Dia: 230 nm Height: 1.5 µm Pitch: 390 nm	532 nm	Rhodamine 6G	300	2019	[120]
Au@SiO_2_	Nanopilllar	Dia: 100 nm Pitch: 200 nm	800 nm	IRDye-800cw-labelled goat antihuman IgG	910	2019	[130]
Au	nanoantenna	Dia: 76 nm Height: 50 nm	633 nm	Alexa 647	1100	2013	[119]
Au	bowtie nanoantenna	-	780/820 nm	TPQDI	1340	2009	[50]
Au	D2PA nanoantenna	Dia: 100 nm Height: 65 nm Pitch: 200 nm	785 nm	ICG, IgG	2970, 7400	2012, 2012	[121,122]

In summary, the fluorophores coupling with the extreme EM fields of the LSPs can enhance the intensity of fluorescence emission up to several orders of magnitude. The “hotspot” region demonstrated the highest MEF enhancements, with a single fluorophore. Several researchers have reported >10^3^ MEF-EF for various nanostructure configurations with a combination of fluorophores with low quantum yield [121,122].

### 2.4. Metal Enhanced Fluorescence-Based Biosensors Applications

Over the decades, an increasing number of studies have reported implementing MEF-based sensing with pre-established technologies, i.e., fluorescence microscopy, fluorescence microarray scanners, microplate readers, or with new devices developed for fluorescence signal amplification. Combining the plasmonic nanostructures with immunoassays or microarrays offers the unique advantages of detectability, and introduces a wide range of fluorescence-based applications with a large variety of commercially available analytes, as summarized in Table 5. Various detection analytes, including biomarkers, pathogens, and toxins, have been reported in the literature with new detection methodologies and enhanced detection limits to provide a valuable tool for early diagnosis [131,132], point-of-care (POC) diagnosis [133,134], and forensic applications [122,135]. Metallic nanoparticles with a silica spacer and a silica core were reported for quantitative detection of the prostate-specific antigen (PSA) with high sensitivity [133]. The detection antibody was attached to a 50 nm Ag particle labeled with RuBpy dye to monitor the fluorescence intensity associated with the binding event. The reported detection time was 30 min for the binding event with a detection limit of 0.20 ng/mL [133]. In another study, the metallic vertical nanorods were used for the quantitative detection of human semen and vaginal fluids [135]. The sensor chip with Ag-nanorod structures was spotted with the semenogelin-2 antibody and anti-17 beta-estradiol antibody and blocked with 15% dry milk and 85% 1× PBS solution. For detection, the daylight-conjugated protein sample was incubated and reacted with the antibodies and washed after 1-h incubation. The sensor chip provides a semen and vaginal fluid detection limit as low as 0.06 µg/mL and 0.005 µg/mL, respectively [135]. In similar studies, a sensor chip was developed for detecting the severe acute respiratory syndrome-coronavirus (SARS-CoV) proteins [134] and swine-origin influenza A (H1N1) viruses (S-OIV) [131] using the LSPs fluorescence method. The detection limits of 0.1 pg/mL for the SARS-CoV N protein [134], and 13.9 pg/mL for S-OIV [131] have been documented.

Immunoassay’s fluorescence detects the target analytes in the buffer solution by selective capturing of the biomarker with tags immobilized over the sensor surfaces. Zhou et al. [122] have reported the D2PA nanoantenna structures and a molecular spacer to enhance fluorescence intensity of protein A immunoassay and human IgG. The detection limit of 0.3 fM (1 × 10^−7^ nM) was reported with a detection time of 1 h. In another study, Zhang et al. [130] have reported on the EBOV immunoassay sensor for the detection of EBOLA virus using 3D plasmonic nanoantenna arrays. The detection limit of ≈220 fg/mL, which was ≈240,000-fold higher than the existing FDA recommended EBOV-rapid-immunoassay.

In the current scenarios of the COVID-19 pandemic, rapid and real-time detection is desirous. Ganguli et al. [136] reported a fluorescence-based sensing platform with a detection limit of 50 RNA copies/µL in the viral transport medium solution, and 5000 RNA copies/µL in the nasal solution. The rapid detection has been demonstrated within 40 min, which makes fluorescence-based detection a viable solution for mass-testing in the current situation.

**Table 5:** Overview of metal enhanced fluorescence-based biosensors for the detection of various analytes, with information on detection limit, detection time, and publication information such as year and reference, for further reading.

Detection Analyte	Detection Time	Limit of Detection	Year	Ref.
Mouse IgG antigen	-	0.25 µg/mL	2015	[132]
Human Semen	60 min	0.06 µg/mL	2018	[135]
Human Vaginal Fluid	60 min	0.005 µg/mL	2018	[135]
Human immunoglobulins	60 min	0.0008 µg/mL	2019	[137]
FITC-labeled YebF protein from Escherichia coli	-	17.2 ng/mL	2020	[138]
Prostate-Specific Antigen (PSA)	30 min	0.20 ng/mL	2017	[133]
S-OIV	-	13.9 pg/mL	2010	[131]
17-β-estradiol	Real-time	1 pg/mL	2017	[139]
SARS-CoV	-	1 pg/mL	2009	[134]
Kidney injury molecule-1	-	500 fg/mL	2018	[104]
Ebola virus	10 s	220 fg/mL	2019	[130]
Neutrophil gelatinase-associated lipocalin	-	0.5 fg/mL	2018	[104]
DNA-oligonucleotides	-	2.5 × 10^4^ nM	2015	[36]
CD4-mRNA expression	60 mints	125 nM	2019	[140]
Glucose	Real-time	50 nM	2015	[141]
Intracellular Adenosine triphosphate	-	35 nM	2020	[96]
Lysozyme in Human Serum	Real-Time	1.6 nM	2020	[101]
Human Immunoglobulin G	-	10 nM	2014	[79]
Carbohydrate-lectin	5 s	0.87 nM	2015	[111]
DNA aptamer	20 min	0.33 nM	2015	[142]
Acetylcholinesterase	-	0.01 nM	2018	[143]
Streptavidin	10 min	0.05 nM	2011	[144]
Hairpin *ssDNA*	30 min	10 pM	2017	[80]
miRNA-21-Bladder cancer-related biomarker in Urine	120 min	26.3 fM	2019	[145]
Human NOGGIN	25 µs	1.5 × 10^−3^ nM	2018	[146]
Alexa 488 labelled oligonucleotide	-	1 × 10^−5^ nM	2016	[106]
Human IgG	60 min	1 × 10^−7^ nM	2012	[122]

### 2.5. Summary and Future Outlook

The continuous exploration of MEF underlying principles and multiple fabrication approaches has increased the success factor in plasmonic nanostructures research. During the past decades, various nanostructures based MEF platforms have been developed and applied in the field of biotechnology and life sciences. These have added the extra features of incident light confinement, spectral properties tunability, enhanced electromagnetic field, and improved signal-to-noise ratio to MEF platforms due to their geometries. This article critically reviews the fabrication methods, material selection, and dimensional features of nanostructures, which can significantly enhance the EF and sensing accuracy. It has been observed that each fabrication method exhibits significant MEF performance due to morphological-specific features. For example, metallic core-shell nanoparticles fabricated by chemical synthesis methods [97,100] have demonstrated enhancements beyond the MEF standard approach through controlling the cavity, which concentrates the electromagnetic field [13]. Similarly, the localization of the electromagnetic field increases with an increase in the length of nanostructures when fabricated with deposition methods [23,109].

**Figure 6:**
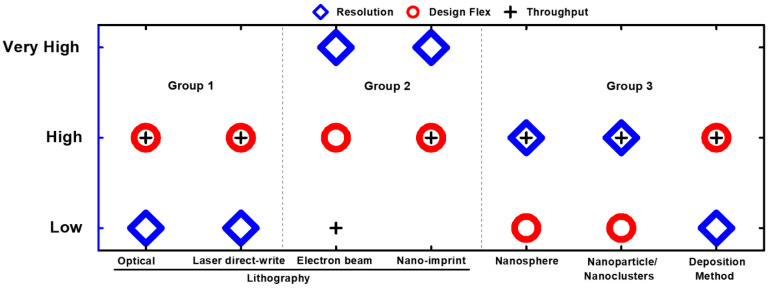
A qualitative comparison of characteristics belonging to different methods for fabricating the nanostructures.

Despite the continuous enhancement in the MEF factor, significant challenges still need to be resolved in order to achieve widespread usability of MEF-based technology, and to reach its full potential. One of the potential research domains is to predict the effect of the dielectric medium and nanostructures geometry on the MEF performance. Maxwell’s equation-based numerical simulations have the potential to evaluate the structure-based performance. However, due to the complexity of its nature, there is a need for accurate and straightforward methods to predict and optimize structural performance. Dipole–dipole coupling methods could be another option, but they have not yet been fully explored for MEF-based applications. Another research domain is the fabrication of large-area MEF substrates with uniform structural features, i.e., size, shape, and distribution with control, precision, and repeatability at the nanoscale resolution, to govern spectral properties. When designing the nanostructures, a combination of appropriate material selection and state-of-art fabrication methods is desired. A brief comparison of fabrication methods in terms of resolution, design flexibility, and throughput is illustrated in Figure 6.

Another potential research domain is the development of new fabrication technologies to develop nanostructures with high throughput, ultrahigh resolution, and design flexibility in an economical way. Chemical synthesis and deposition methods presently provide marginal accuracy with high throughput for nanostructures fabrication, while lithography methods are expensive to apply for large-area applications. Therefore, new fabrication approaches, combining the existing techniques, are required to fabricate highly sensitive biosensors for widespread MEF-based applications. Nanoimprinting lithography (NIL) is notable among other reported methods due to its potential for controlling the nano-feature size with high throughput and high resolution [122]; it was adapted to achieve an enhancement factor on the order of > 10^3^. Such a giant MEF enhancement has made the fabricated platform capable of detecting 0.3 fM (1 × 10^−7^ nM) human IgG. Recently, Zang et al. [130] reported on 3D nanoantenna structures fabricated by combining advanced nanoimprinting and deposition methods with precise dimensional control, for detecting the EBOLA virus with improved sensitivity of 240,000-fold compared to the FDA-recommended EBOV immunoassay sensor. The advancement has paved the way for future developments required for early diagnostics of diseases. Recently, SARC-CoV-2 genome RNA detection was demonstrated for real-time-point of diagnostics using the fluorescence-based portable platform with a detection limit of 5000 RNA copies/µL. However, further developments are required to accelerate rapid and real-time detections. These developments would collectively improve the fabrication of highly sensitive and portable platforms, which will help to stimulate future developments for real-time diagnostics of SARC-CoV-2 by providing a substitute for the laboratory-based test.

## Figures and Tables

**Table 1:** Characteristics of various metals used for metal-enhanced fluorescence (MEF) applications.

Metals	Plasmonic Characteristics					Chemical Reactivity	Reference
	UV	VIS	NIR	IB	Q Factor		
Silver (Ag)		-	-		High	Biocompatible; easily oxidized	[12]
Copper (Cu)				- <600 nm	Low	Easily oxidized	[13,16]
Gold (Au)		-	-	- <500 nm	High	Biocompatible; Stable	[18,19]
Aluminium (Al)	-	-			Low	Stable after surface passivation	[17]
Palladium (Pd)		-			Low	Stable	[14,15]
Platinum (Pt)		-			Low	Stable	[15]

UV: ultraviolet; VIS: visible, NIR: near-infrared; IB: inter-band; Q factor: quality factor.

**Table 3:** Summary of various nanostructures fabricated via various deposition methods with different shapes and geometries, and their experimentally determined enhancement factor (EF), with feature size, excitation wavelengths, used fluorophores, and publication information such as year and reference, for further reading.

Material	Configuration of Structures	Structures Feature Size (nm)	Wavelength λ (nm)	Fluorophore	EF	Year	Ref.
Cu	Structured thin-film nanorods	Height: 550 nm	590 nm	Rhodamine 123	02	2012	[17]
Au	Structured thin-film nanorods	Dia: 40 nm Height: 285 nm	590 nm	Rhodamine 123	3.9	2012	[17]
Zno	Vertical nanorods	Dia: 83.2 nm Height: 170 nm	645 nm	Alexa Fluor 647	5.7	2015	[110]
Au	Nanorods	Dia: 30 nm Height: 13 nm	650 nm	Alexa 647	10	2015	[111]
Ag	Structured thin-film nanorods	Dia: 75 nm Height: 400 nm	590 nm	Rhodamine 123	20	2011	[112]
Ag	Slanted nanorods	Height: 1000 nm	635 nm	Cy5	23	2013	[108]
Ag	Structured thin-film nanorods	Dia: 75 nm Height: 400 nm	590 nm	Rhodamine 123	23	2012	[17]
ZnO	Flower shape nanorods	Dia: 718.5 nm Height: 200 nm	515 nm	Alexa Fluor 532	25	2015	[110]
Ag	Zigzag nanorods	Height: 2000 nm	525 nm	Alexa 488	28	2016	[106]
Ag	Nanocone	Dia_base_: 180 nm Height: 500 nm	528 nm	Rhodamine 6G	30	2011	[113]
Ag	Slanted nanorods	Length: 635 nm	555 nm	Rhodamine 6G	32	2015	[109]
Al	Structured thin-film nanorods	Dia: 30 nm Height: 1000 nm	590 nm	Rhodamine 123	37	2012	[17]
Zno	Flower shape nanorods	Dia: 718.5 nm Height: 200 nm	645 nm	Alexa Fluor 647	45	2015	[110]
Ag	Structured thin-film nanorods	Dia: 75 nm Height: 400 nm	590 nm	Rhodamine 123	71	2012	[17]
Ag	Slanted nanorods	Dia: 220 nm Height: 3000 nm	-	Bovine aortic endothelial cell	-	2010	[114]
Ag	Vertical nanorods structures	Dia: 120 nm Height: 500 nm	635 nm	Cy5	200	2018	[22]
Ag	nanorods	Dia: 89 nm Height: 3000 nm	520 nm	fluorescein-5-isothiocyanate	494	2014	[115]
